# Exposure to the Proton Scavenger Glycine under Alkaline Conditions Induces *Escherichia coli* Viability Loss

**DOI:** 10.1371/journal.pone.0060328

**Published:** 2013-03-27

**Authors:** Donna Vanhauteghem, Geert Paul Jules Janssens, Angelo Lauwaerts, Stanislas Sys, Filip Boyen, Eric Cox, Evelyne Meyer

**Affiliations:** 1 Department of Nutrition, Genetics and Ethology, Faculty of Veterinary Medicine, Ghent University, Merelbeke, Belgium; 2 Department of Pharmacology, Toxicology and Biochemistry, Faculty of Veterinary Medicine, Ghent University, Merelbeke, Belgium; 3 Taminco, Ghent, Belgium; 4 Department of Internal Medicine and Clinical Biology of Large Animals, Faculty of Veterinary Medicine, Ghent University, Merelbeke, Belgium; 5 Department of Pathology, Bacteriology and Avian Diseases, Faculty of Veterinary Medicine, Ghent University, Merelbeke, Belgium; 6 Department of Virology, Parasitology and Immunology, Faculty of Veterinary Medicine, Ghent University, Merelbeke, Belgium; Centre National de la Recherche Scientifique, Aix-Marseille Université, France

## Abstract

Our previous work described a clear loss of *Escherichia coli* (*E. coli*) membrane integrity after incubation with glycine or its *N*-methylated derivatives *N*-methylglycine (sarcosine) and *N,N*-dimethylglycine (DMG), but not *N,N,N*-trimethylglycine (betaine), under alkaline stress conditions. The current study offers a thorough viability analysis, based on a combination of real-time physiological techniques, of *E. coli* exposed to glycine and its *N*-methylated derivatives at alkaline pH. Flow cytometry was applied to assess various physiological parameters such as membrane permeability, esterase activity, respiratory activity and membrane potential. ATP and inorganic phosphate concentrations were also determined. Membrane damage was confirmed through the measurement of nucleic acid leakage. Results further showed no loss of esterase or respiratory activity, while an instant and significant decrease in the ATP concentration occurred upon exposure to either glycine, sarcosine or DMG, but not betaine. There was a clear membrane hyperpolarization as well as a significant increase in cellular inorganic phosphate concentration. Based on these results, we suggest that the inability to sustain an adequate level of ATP combined with a decrease in membrane functionality leads to the loss of bacterial viability when exposed to the proton scavengers glycine, sarcosine and DMG at alkaline pH.

## Introduction

We recently described a decrease in the viability of stationary phase enterotoxigenic *Escherichia coli* (*E. coli, ETEC*) associated with membrane damage and reduced growth capacity caused by glycine and its *N*-methylated derivatives *N*-methylglycine (sarcosine), *N,N*-dimethylglycine (DMG) under alkaline stress conditions [1]. In contrast, the trimethylated analogue of glycine, betaine, did not affect bacterial viability. We now aim to investigate which changes in viability parameters accompany this selective loss of membrane integrity at alkaline pH, providing an indication on the possible underlying mechanism.

**Figure 1:**
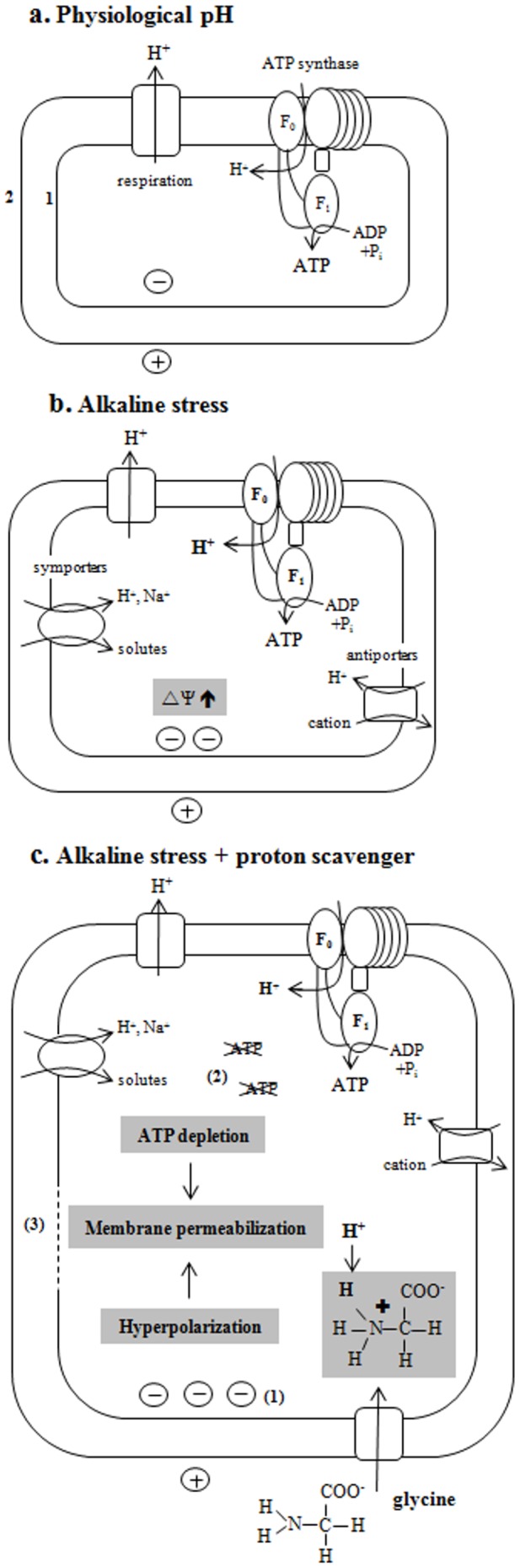
Maintaining *E. coli* pH homeostasis at physiological pH (a). During respiration, protons (H^+^) are pumped extracellularly, while ATP synthesis via the F_o_F_1_-ATP synthase complex moves protons intracellularly. F_o_F_1_-ATP synthase converts the free energy of the proton motive force (PMF) into the chemical energy source ATP. Under physiological conditions, the extracellular pH is more acid than the intracellular pH. The cytoplasmic membrane is negatively charged on the inside, and positively charged on the outside. (1) represents the cytoplasmic membrane, (2) represents the outer membrane. **Maintaining** ***E. coli*** **pH homeostasis under alkaline stress conditions (b).** To prevent cytosolic alkalinisation under extracellular alkaline conditions, the cytoplasmic pH is-next to other mechanism - also regulated by the import of protons by the upregulated ATP synthase and by a multitude of cation antiport systems, pumping in protons. The membrane potential (▵Ψ) is relatively increased (i.e. more negative) to compensate for the inverted proton concentration gradient (▵pH). **Exposure of alkaline stressed** ***E. coli*** **to proton scavenging amines such as glycine (c)**. When unprotonated glycine enters the neutral cytosol under extracellular alkaline conditions it becomes protonated. This causes membrane hyperpolarisation (1) by proton consumption and a higher ATP consumption in an effort to sustain pH homeostasis (2). These effects induced by proton scavenging lead to a loss of membrane integrity (3).

Direct membrane interactions of peptides and amino acid-based surfactants causing antibacterial effects are usually related to a net positive charge of these compounds, enhancing their interaction with anionic lipids and other bacterial targets [2]. However, antibacterial effects through membrane altering actions of anionic amino acid-based surfactants have also been described [3], [4]. Besides a direct cytoplasmic membrane effect, it is also possible that the ETEC membrane damage occurs secondary to a negative influence on bacterial physiology [5]. Indeed, alkaline stress affects bacterial homeostasis mechanisms [6], enhancing ETEC susceptibility to a disturbance of their functional integrity by glycine, sarcosine and DMG. Figure 1a represents the physiological conditions (neutral, i.e. no pH stress), where the intracellular pH is kept in a narrow range near pH 7.6 [7], [8]. A shift to an alkaline environment (Fig. 1b) is stressful for bacteria as illustrated by the induction of major stress systems in *E. coli*, such as heat shock responses [9], [10], the SOS regulon [11], and the Cpx envelope stress mechanisms [12]. pH stress also induces several physiological changes [13] and initiates a large number of adaptive strategies. These strategies include: (i) increased metabolic acid production through amino acid deaminases and sugar fermentation; (ii) increased ATP synthase that couples H^+^ entry to ATP generation; (iii) changes in cell surface properties; and (iv) increased expression and activity of monovalent cation/proton antiporters [6], [7]. Interference at various levels of the compensation mechanisms may eventually lead to a loss of bacterial viability.

Different complementary approaches can be used to assess bacterial viability/activity [14], [15], [16], [17]. In our previous work we established the loss of membrane integrity due to exposure to glycine, sarcosine and DMG under alkaline conditions [1]. These experiments were repeated using log phase *E. coli* and the occurrence of membrane damage was further assessed by measuring the leakage of nucleic [18]. Traditional culture based methods, minimal inhibitory concentrations (MIC) and plate counts, were also performed. The energy status of bacteria is an important functional indicator of bacterial viability. Bacteria use two forms of metabolic energy: electrochemical energy provided by ion gradients and energy-rich phosphate bonds, such as ATP [14]. Measurements of these forms of energy, such as membrane potential and ATP concentration can be used as indicators of cell viability [19]. Next to ATP, the inorganic phosphate (polyP) concentration is also related to the energetic status of bacteria as polyP is considered to be a general source of ATP in several well-known reactions [20]. Moreover, polyP has also been found to be involved in several bacterial stress responses [20], [21]. Closely connected to the energy metabolism of *E. coli* is its respiratory activity, as the electro-chemical gradient of protons generated by respiration in the process of oxidative phosphorylation is used to synthesize ATP from ADP and inorganic phosphate [22], [23]. Both the respiratory chain and F_o_ F_1_-ATP synthase have been reported to regulate the intracellular pH in bacteria [24], [25]. General enzyme activity is another essential factor in maintaining cellular viability. For this purpose, esterases can provide a good indication of the bacterial metabolic activity. They demonstrate the cells' capacity to synthesize these enzymes and maintain them in an active form [17].

The present study performed an in-depth viability analysis based on a combination of real-time physiological techniques which generated novel data allowing the formulation of a clear hypothesis on the effects of glycine, sarcosine and DMG on *E. coli* under alkaline stress conditions, as illustrated in Fig. 1.

## Materials and Methods

### Test Compounds

Glycine (≥99% purity), sarcosine (≥99% purity) and betaine (≥99% purity) were obtained from Sigma Aldrich (St. Louis, MO), DMG (≥97% purity) was obtained from Taminco (Taminco B.V.B.A., Ghent, Belgium). All compounds were stored to manufacturers' guidelines until use. Compound characteristics are described by Vanhauteghem *et al.* [1].

### Bacterial strain, culture and exposure conditions

The haemolytic ETEC strain GIS26, serotype 0149:F4ac, positive for heat labile (LT) and heat stabile (STa and STb) enterotoxins [26], was grown overnight at 37°C in brain heart infusion medium (BHI, Oxoid Limited, Hampshire, United Kingdom) to stationary phase. This overnight culture was inoculated 1∶100 into fresh BHI broth and grown for 3 h to the exponential phase at 37°C. Log phase bacteria were collected by centrifugation (10.000× g, 2 minutes, room temperature) of 1 ml of the bacterial culture and then resuspended in each of the appropriate test compound solutions in sterile PBS.

A 50 mM solution of each test compound was prepared in sterile PBS. The pH of these solutions was adjusted to pH 9.5 by either HCl or NaOH addition. Log phase ETEC were dispersed in 1 ml of each test sample. As a control, pH-adjusted sterile PBS was used. The bacterial suspensions were incubated shaking for 5, 15, 30, 90 and 180 minutes at 37°C. After incubation, analysis of the different viability parameters was performed as described for each parameter below. Proper positive and negative controls were included for each analysis.

### Minimum inhibitory concentration (MIC)

Determination of the minimum inhibitory concentration (MIC) for the test compounds was performed using the broth microdilution assay, using Mueller-Hinton agar at a pH of 6.5, 8.5, 9.0, 9.5 and 10.0. Inoculated microwells free of the test substance, but adjusted to the respective pH values, were included as growth controls, uninoculated microwells were used as sterility controls. Results were recorded after 20 h incubation of the microwell plates in an aerobic atmosphere at 35°C (+/−2°C).

### Plate counts

The number CFU per ml was assessed by conventional plate count, which is based on CFU values obtained from a 10-fold serial dilution of each sample plated on Tryptone Soy Agar (Oxoid Limited, Hampshire, United Kingdom) and incubated overnight at 37°C. These plate counts determine the number of culturable bacteria in each sample. All data are the result of triplicate experiments.

### Leakage of 260-nm-absorbing material

After incubation samples were centrifuged at 10.000 g for 2 min at 4°C, and 750 µl of the supernatant for each treatment was added to quartz cuvettes and absorbance values at 260 nm were recorded using a spectrophotometer (Genesys 10UVn Thermo Electron Company, Cambridge, UK). All experiments were performed in triplicate.

### Flow cytometric parameters

All data were obtained using a FACSCanto flow cytometer (Becton, Dickinson and Company, Erembodegem, Belgium), and acquired and processed using FacsDiva software (Becton, Dickinson and Company, Franklin Lakes, NJ, USA). All experiments were performed in triplicate.

**Membrane integrity** was assessed using the LIVE/DEAD BacLight^TM^ kit (Molecular Probes Eugene, OR, USA) as described by the manufacturer. This bacterial viability kit is widely used in flow cytometry and consists of two nucleic acid stains: green fluorescent SYTO 9 is cell-permeable and freely enters all ETEC, either live or dead, while red fluorescent propidium iodide (PI) can only enter membrane-comprised cells [27]. In our set-up, 10 µl of the treated bacterial cell suspension was added to 987 µl of sterile saline. These samples were immediately stained with 3 µl of a mixture of SYTO 9 (5 µM final concentration) and PI (30 µM final concentration) and incubated for 15 minutes in the dark at room temperature. Flow cytometric measurements were performed immediately thereafter.

**Membrane potential** was assessed using the BacLight^TM^ Membrane Potential kit (Molecular Probes Eugene, OR, USA) as described by the manufacturer. The kit contains DiCO_2_ which exhibits green fluorescence in all bacterial cells, but the fluorescence shifts toward red emission as the dye molecules self-associate at the higher cytosolic concentrations caused by larger membrane potentials. The red to green fluorescence ratio is used as a size-independent indicator for membrane potential. The proton ionophore carbonyl cyanide m-chlorophenylhydrazone (CCCP) was used to provide a depolarized control as it destroys membrane potential by eliminating the proton gradient. In our set-up, 10 µl of the treated bacterial cell suspension was added to 980 µl of sterile PBS. These samples were immediately stained with 10 µl of a DiOC_2_ (30 µM final concentration) and incubated for 30 minutes at 37°C in the dark. Flow cytometric measurements were performed immediately thereafter.

**Esterase activity** was assessed using 5(6)-carboxyfluorescein diacetate (cFDA, Molecular Probes, Eugene, OR, USA). cFDA is an esterified fluorogenic substrate widely used for assessing esterase activity in bacteria. It is cell permeant and once inside the cell, the non-fluorescent cFDA is enzymatically cleaved via hydrolysis of the diacetate groups by nonspecific esterases into fluorescent carboxyfluorescein (cF), which is accumulated cytosolically [28]. Esterified fluorogenic substrates offer a means of rapid detection of metabolically active bacteria when used in combination with techniques that measure fluorescence at the single cell level, such as flow cytometry. Prior to use, concentrated stock solutions of 21.7 mM cFDA were prepared in DMSO, and further diluted to a final concentration of 10 mM in sterile PBS. Samples of 1 ml of the treated bacterial suspensions were centrifuged (10.000× g, 2 minutes) and the supernatant was discarded. Cell pellets of ETEC were resuspended in 20 µl of 10 mM cFDA and incubated for 30 min at 37°C in the dark. Following incubation, cells were washed and resuspended in 1 ml sterile PBS and then analyzed by flow cytometry.

**Respiratory activity** was assessed using 5-cyano-2,3-ditolyl tetrazolium chloride (CTC). CTC is a colourless, membrane-permeable compound that produces a red fluorescing precipitate in the cell when it is reduced to its formazan by the electron transport system of bacterial cells [29]. In our set-up, 20 µl of the treated bacterial cell suspension was added to 880 µl of sterile PBS. These samples were immediately stained with 100 µl of a CTC (5 µM final concentration) and incubated for 30 minutes at 37°C in the dark. Flow cytometric measurements were performed immediately thereafter.

### ATP measurement

For the determination of total ATP, the BacTiter-Glo^TM^ System (Promega, Madison, WI, USA) was used as described by the manufacturer. The BacTiter-Glo^TM^ Buffer was mixed with the lyophilized BacTiter-Glo^TM^ Substrate and equilibrated at room temperature. The mixture was stored for 3 h at room temperature to ensure that all ATP was hydrolysed (“burned off”) and the background signal had decreased. A test sample of 100 µl i.e. both the bacterial population and the incubation medium, was taken from the exposed resuspended bacterial populations and mixed with an equal volume of BacTiter-Glo^TM^ Reagent. Luminescence was measured with a multiplate luminometer (Fluoroscan Ascent FI, Thermo Labsystems, Helsinki, Finland). A calibration curve with adequate dilutions of pure rATP (Promega, Madison, WI, USA) was measured for each buffer prepared. All data are the result of triplicate experiments.

### Inorganic phosphate (polyP) measurement

Intracellular polyP was measured in cell suspension using the DAPI-based fluorescence approach [30]. Cells were washed and resuspended in buffer T (100 mM Tris HCl, pH 7.5) and DAPI (4′6-diamidino-2-phenylindole) (Sigma Aldrich, St. Louis, MO) was added to a final concentration of 10 µM. After 5 min agitation at 37°C, the DAPI fluorescence spectra (excitation 420 nm with a bandwith of 8 nm, emission 535 nm with a bandwith of 25 nm) were recorded using a Perkin Elmer Envision Xcite spectrofluorometer. The fluorescence of the DAPI-polyP complex was used as a measure of intracellular poly P because fluorescence emissions from free DAPI and DAPI-DNA are minimal at this wavelength [30]. All data are the result of triplicate experiments.

### Statistical analysis

Flow cytometric data of the percentages of live, intermediate and dead bacteria were arcsine-transformed to obtain normal distributions. The CFU counts were logarithmically transformed. ATP, polyp, membrane potential, respiratory activity and leakage at 206 nm data were not transformed. In order to compare the effects of the compounds with the control condition, the Welch' robust variation of ANOVA was used and followed by Dunnett's T3 multiple comparisons. Covariance analysis was performed to compare linear regression slopes of the time course of CFU counts, ATP concentration and flow cytometric based membrane integrity analysis for the different compounds versus control conditions.

## Results

### MIC determination

Concentrations ranging from 25 mM up to 200 mM of each test compound were investigated for their antibacterial potential against ETEC at a pH ranging from 6.5 to 10.0. None of the tested compounds inhibited visible bacterial growth at pH 6.5 to pH 9.0. In contrast, at pH 10.0 all bacterial growth was inhibited due to the highly alkaline pH, as no bacterial growth was observed in the control sample either. At pH 9.5 compound- and concentration-dependent effects occurred. While no inhibition of bacterial growth was yet seen due to exposure to sarcosine, DMG or betaine, glycine inhibited the ETEC growth at a concentration of 200 mM. This indicates that the MIC for glycine on the ETEC at pH 9.5 lies between 100 and 200 mM, while it exceeds 200 mM for the *N*-methylated analogues tested. The MIC data are presented in Table 1.

**Table 1:** Overview of the MIC determination data for glycine, sarcosine, DMG and betaine.

			Concentration			
pH	treatment		25 mM	50 mM	100 mM	200 mM
6.5	glycine		+	+	+	+
	sarcosine		+	+	+	+
	DMG		+	+	+	+
	betaine		+	+	+	+
	control		+	+	+	+
8.5	glycine		+	+	+	+
	sarcosine		+	+	+	+
	DMG		+	+	+	+
	betaine		+	+	+	+
	control		+	+	+	+
9.0	glycine		+	+	+	+
	sarcosine		+	+	+	+
	DMG		+	+	+	+
	betaine		+	+	+	+
	control		+	+	+	+
9.5	glycine		+	+	+	−
	sarcosine		+	+	+	+
	DMG		+	+	+	+
	betaine		+	+	+	+
	control		+	+	+	+
10.0	glycine		−	−	−	−
	sarcosine		−	−	−	−
	DMG		−	−	−	−
	betaine		−	−	−	−
	control		−	−	−	−

The inhibition of visible bacterial growth by concentrations ranging from 25 mM up to 200 mM of each test compound was investigated at a pH ranging from 6.5 to 10.0. The symbol (+) represents visible bacterial growth, while the symbol (−) represents no visible growth.

### Plate counts

**Figure 2:**
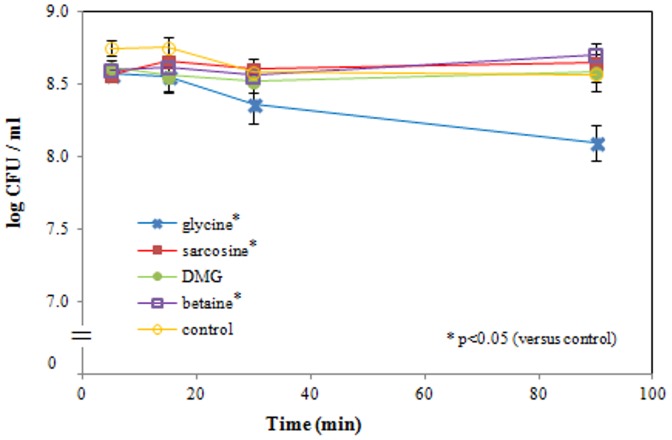
Culturability of *E. coli*. ETEC were exposed to glycine, sarcosine, DMG, betaine (50 mM, pH 9.5) and sterile PBS (control, pH 9.5) for up to 90 minutes. The slope of the time course of the glycine-, sarcosine- and DMG-exposed samples is significantly more negative than the slope of the control sample. In contrast, the slope of the betaine exposed sample is more positive compared to the control sample. Data are expressed as means ± SD of triplicate experiments.

Complementary to the standard evaluation method of bacterial growth inhibition via the above-described MIC, the bacterial culturability was also assessed through plate counts. Mean values ± SD of log phase ETEC are presented in Fig. 2. Covariance analysis showed a significant difference in linear regression of the control versus glycine, sarcosine and betaine. The slope of the time course of the glycine- and sarcosine-exposed samples was significantly more negative than the slope of the control sample (p = 0.002 and 0.003, respectively). In contrast, the slope of the betaine-exposed sample was more positive compared to the control sample (p<0.0005).

### Leakage of 260-nm-absorbing material

**Figure 3:**
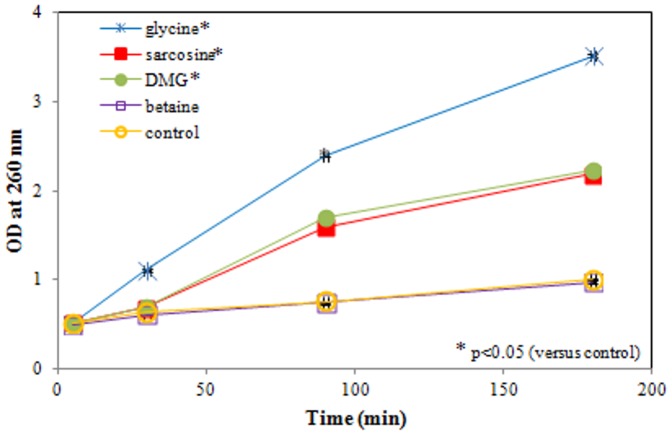
Leakage of 260-nm-absorbing material from *E. coli*. ETEC were exposed to glycine, sarcosine, DMG, betaine (50 mM, pH 9.5) and sterile PBS (control, pH 9.5) for up to 180 minutes. After 30 min of incubation there is a significant loss of 260-nm-absorbing material when incubated with glycine (p = 0.003) and DMG (p = 0.006). Exposure to sarcosine leads to a significant increased leakage after 90 min of incubation (p = 0.001). Data are expressed as means ± SD of triplicate experiments.

The presence of nucleic acids and its related compounds, such as pyrimidines and purines, are used as an indicator of cell membrane damage. Absorbance was measured for up to 180 min of ETEC exposure to each of the four test compounds (50 mM, pH 9.5), and compared to the control. Mean optical density (OD) values ± SD are presented in Fig. 3. After 30 min of incubation there was a significant loss of 260-nm-absorbing material when incubated with glycine (p = 0.003) and DMG (p = 0.006). Exposure to sarcosine also led to a significant increased leakage albeit only after 90 min of incubation (p = 0.001). In contrast, betaine did not show any influence on membrane permeability under these incubation conditions. These results confirm a loss of membrane integrity due to exposure to glycine, and to a lesser and slower extent sarcosine and DMG under alkaline conditions.

### Flow cytometric assessment of membrane permeability

**Figure 4:**
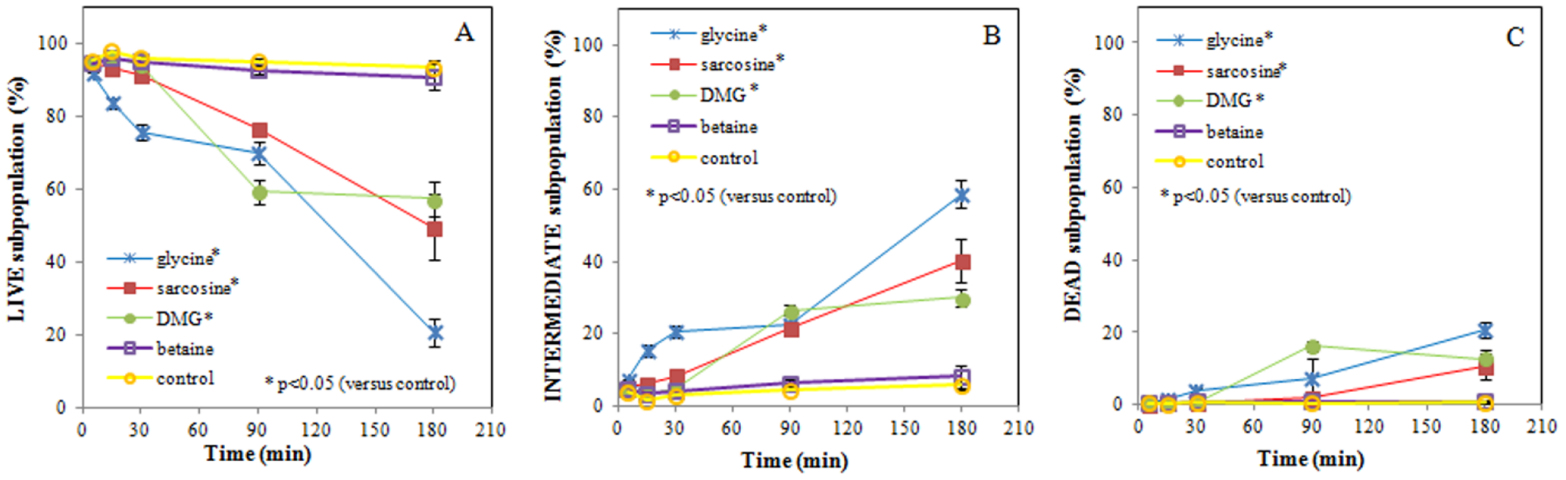
Percentage of membrane-intact live (A), membrane-damaged “intermediates” (B) and irreversibly membrane-damaged dead (C) *E*. *coli subpopulations.* ETEC were exposed to glycine, sarcosine, DMG, betaine (50 mM, pH 9.5) and sterile PBS (control, pH 9.5) for up to 180 minutes Data are expressed as means ± SD of triplicate experiments.

As previously shown for stationary phase ETEC [1], flow cytometric analysis of membrane integrity of log phase ETEC with SYTO 9/PI dual staining revealed a unique fluorescence pattern, directly related to the degree of membrane damage. Three bacterial subpopulations could thus be identified: membrane-intact live bacteria (A), membrane-damaged “intermediates” (B) and dead bacteria (C). Percentages of these bacterial subpopulations are presented in Fig. 4. Data showed a significant (p = 0.001) decrease in the mean percentage of live bacteria already after an exposure time of 5 min to glycine (50 mM, pH 9.5) compared to the control. A significant decrease in the percentage of live bacteria after exposure to sarcosine or DMG was observed only after 90 min of incubation (p = 0.003 and 0.005, respectively).

**Figure 5:**
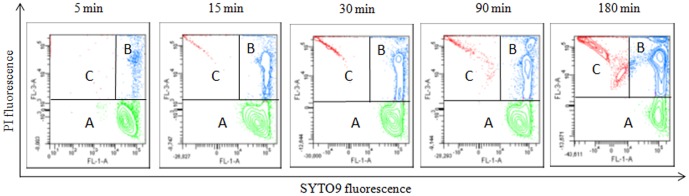
Flow cytometric SYTO 9 (FL1)/PI (FL3) dot plots presenting pH-dependent *E. coli* membrane damage. Data obtained for a representative ETEC sample incubated with glycine (50 mM, pH 9.5) for up to 3 hours. The A region corresponds to the subpopulation of live cells with an intact plasma membrane, the B region corresponds to the subpopulation of bacteria in an intermediate injured state with different degrees of comprised membranes, the C region corresponds to the subpopulation of dead cells with irreversibly damaged membranes.

Covariance analysis showed a significant difference in linear regression of the control versus glycine, sarcosine and DMG. The slope of the time course of the percentages of membrane-intact live bacteria exposed to the latter 3 compounds was significantly more negative than the slope of the control sample (all p<0.0005). Complementary, the slopes of the membrane-damaged intermediate and dead bacteria were significantly more positive compared to the control sample (all p<0.0005). Illustrative flow cytometric dot plots of ETEC samples incubated with glycine (50 mM, pH 9.5) are presented in Fig. 5.

Overall, these results suggest a compound- and time-dependent onset of membrane damage by glycine versus sarcosine and DMG. In marked contrast, no significant differences in ETEC membrane integrity were observed between betaine and the control sample for any of the incubation times measured.

### Flow cytometric assessment of membrane potential

**Figure 6:**
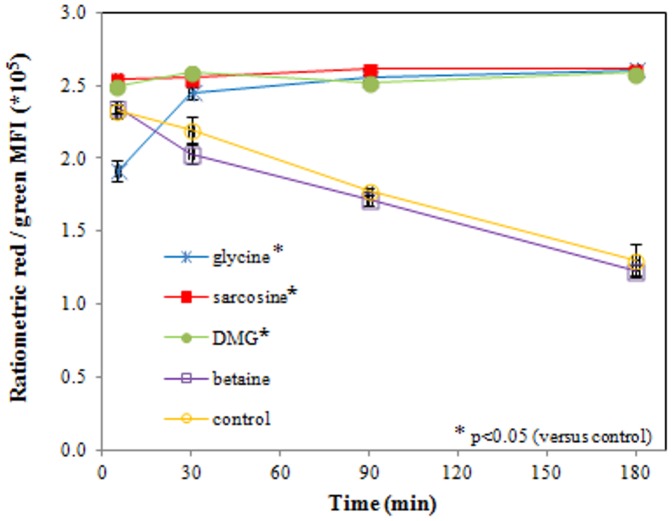
Ratiometric red/green fluorescence presenting *E. coli* membrane potential. Ratiometric membrane potential measurements showed a significant hyperpolarization of the ETEC membrane following exposure to glycine, sarcosine and DMG (50 mM, pH 9.5) after 90 min of incubation, compared to the control (p<0.0005, p = 0.002 and p<0.0005, respectively). In the betaine-exposed and control samples there is a time-dependent depolarization of ETEC. Data are expressed as means ± SD of triplicate experiments.

Ratiometric MFI determination of the membrane potential showed a significant hyperpolarization of the ETEC membrane after exposure to glycine, sarcosine and DMG (50 mM, pH 9.5) after 90 min of incubation, compared to the control (p<0.0005, p = 0.002 and p<0.0005, respectively). In contrast, a time-dependent depolarization of ETEC occurred in the betaine-exposed and control samples. Mean ratiometric MFI values ± SD are presented in Fig. 6.

### Flow cytometric measurement of esterase activity

**Figure 7:**
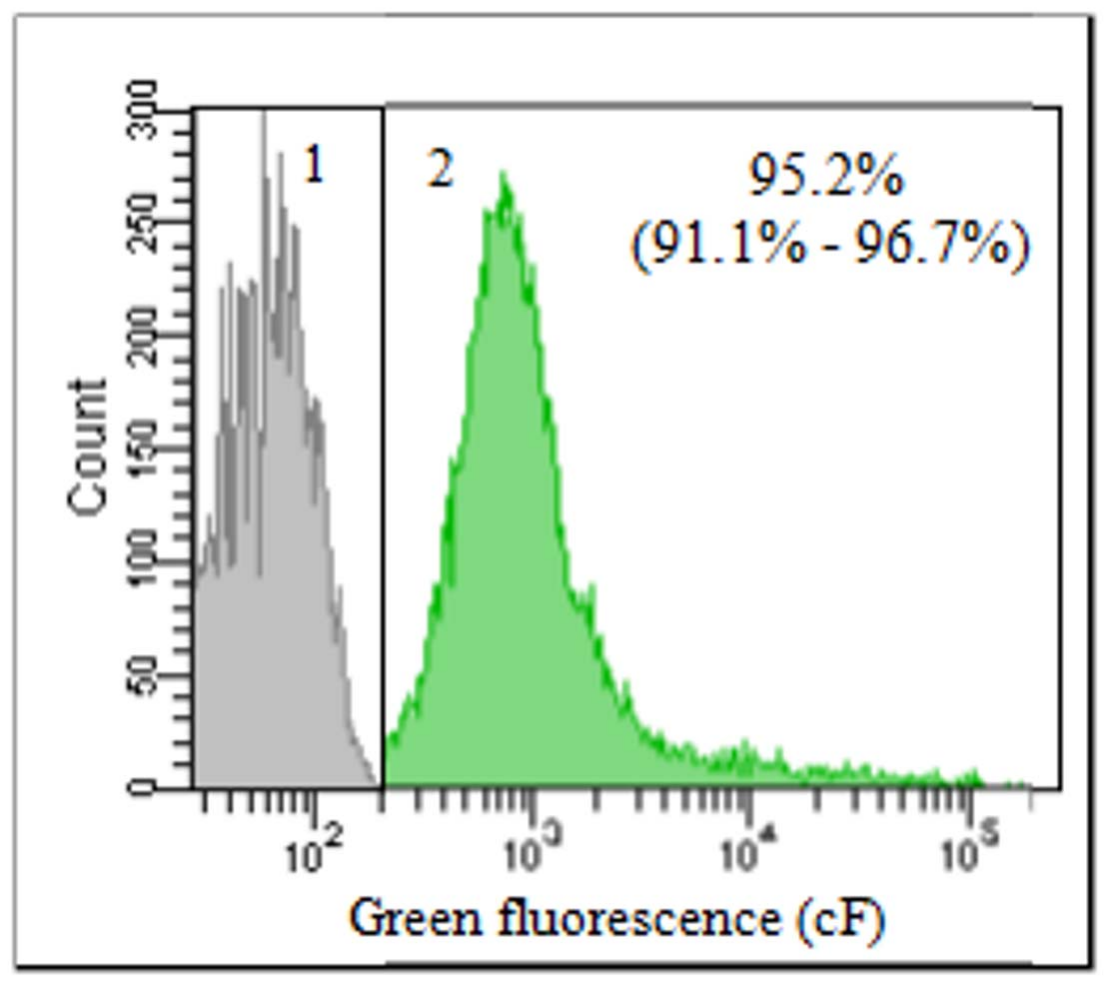
Overlay diagram of the flow cytometric histograms representing metabolically active and inactive *E.* * coli subpopulations.* Inactive (cF^−^) (1) and active (cF^+^) (2) ETEC population are determined by cFDA staining. Inactive cells do not stain because they either lack the enzyme activity to metabolize cFDA to cF and/or cF diffuses freely through the membrane, while metabolically active cells are green fluorescent (cF^+^). Both populations are separated by the black line. The overall mean of triplicates and range are provided.

Esterase activity was measured as a marker for the ETEC metabolic activity. Fig. 7 shows the representative fluorescence pattern of active and inactive control samples. Inactive cells do not stain (cF^−^) because they either lack enzyme activity and/or cF diffuses freely through the membrane. Metabolically active cells are green fluorescent (cF^+^). Compared to the active control, ETEC exposed to only pH 9.5 (control) or to each of the test compounds (50 mM, pH 9.5) showed no decrease in the percentage of metabolically active bacteria for up to 30 min. Overall, these results do not allow to demonstrate a metabolic impairment at the level of enzymatic activity.

### Flow cytometric assessment of respiratory activity

**Figure 8:**
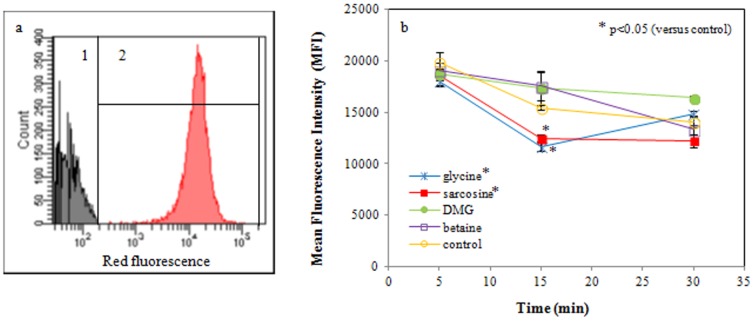
Overlay diagram of the flow cytometric histograms representing respiratory active and inactive *E.* * coli subpopulations (a).* Inactive (1) and active (2) ETEC subpopulation are determined by CTC staining. Inactive cells do not stain because they lack respiratory dehydrogenase activity. Actively respiring cells are red fluorescent. Both populations are separated by the black line. Mean fluorescence intensity (MFI) presenting *E. coli* respiratory activity (b). Compared to the active control, ETEC exposed to glycine and sarcosine (50 mM, pH 9.5) showed a significant decrease in MFI after 15 min of incubation (p = 0.017 and p = 0.008, respectively). Data are expressed as means ± SD of triplicate experiments.

Dehydrogenase activity was measured as a marker for the ETEC respiratory activity. Fig. 8A shows the representative fluorescence pattern of active and inactive control samples. Inactive cells (a) do not stain because they lack respiratory dehydrogenase activity. Actively respiring cells (b) become red fluorescent. Fig. 8B presents the MFI of the bacterial populations after exposure to the compounds. Compared to the active control, ETEC exposed to glycine and sarcosine (50 mM, pH 9.5) showed a significant decrease in MFI already at 15 min of incubation (p = 0.017 and p = 0.008, respectively). This indicates that there is a significant but transient decrease in respiratory activity of the ETEC after 15 min of exposure to glycine and sarcosine at pH 9.5. No significant differences were observed between DMG, betaine and the control sample for any of the incubation times measured.

### ATP Measurement

**Figure 9:**
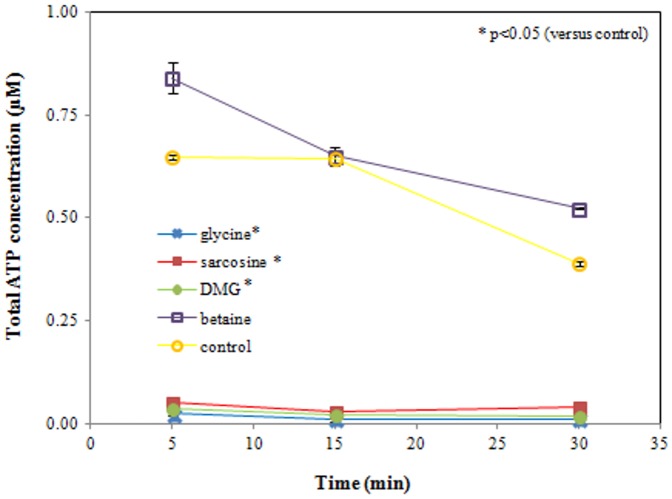
Total ATP concentration of *E. coli*. ETEC were exposed to glycine, sarcosine, DMG, betaine (50 mM, pH 9.5) and sterile PBS (control, pH 9.5) for up to 30 min. Already after 5 min of incubation, a significant (p<0.0005) loss of ATP occurs in the bacterial populations exposed to glycine, sarcosine and DMG, compared to the control and betaine sample. Data are expressed as means ± SD of triplicate experiments.

Intracellular ATP levels were examined for up to 30 min of ETEC exposure to each of the four test compounds (50 mM, pH 9.5), and compared to the control. Mean values ± SD are presented in are presented in Fig. 9.

Cellular ATP depletion started immediately during the incubation with either glycine, sarcosine or DMG. Already after 5 min of incubation a significant (all p<0.0005) loss of ATP was observed in these samples, compared to the control. A time-dependent decrease in the ATP concentration occurred also in the control and betaine samples. However, after 30 min of incubation, the ATP level of the bacterial populations exposed to betaine remained significantly (p = 0.001) higher than the ATP level of the control samples.

### Inorganic phosphate (polyP) measurement

**Figure 10:**
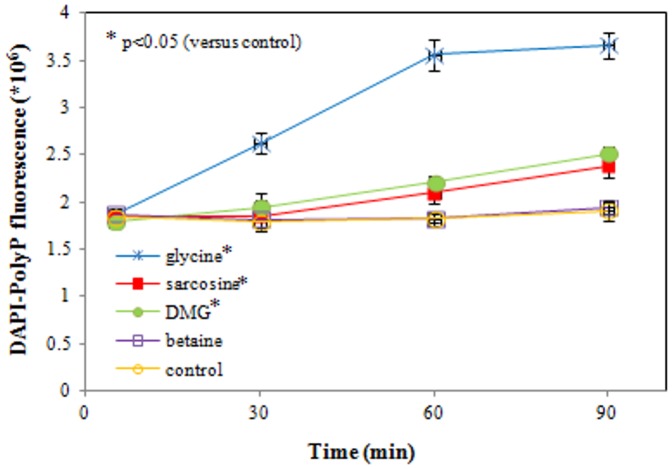
DAPI-polyP fluorescence presenting polyP concentration of *E. coli*. Inorganic phosphate levels were measured for up to 90 min of ETEC exposure to glycine, sarcosine, DMG or betaine (50 mM, pH 9.5), and compared to the control. After 30 min of incubation there is a significant increase in DAPI-polyP fluorescence when incubated with glycine (p = 0.009), compared to the control sample. Exposure to sarcosine and DMG leads to a significant increase of fluorescence after 90 min of incubation (p = 0.03 and p = 0.007, respectively), but not to a level as high as seen after glycine exposure. Betaine exposure had no significant influence compared to the control. Data are expressed as means ± SD of triplicate experiments.

The fluorescence (in arbitrary units) of the DAPI-polyP complex is used as a measure of intracellular polyP. Inorganic phosphate levels were measured for up to 90 min of ETEC exposure to each of the four test compounds (50 mM, pH 9.5), and compared to the control. Mean values ± SD are presented in Fig. 10. After 30 min of incubation there was a significant increase in DAPI-polyP fluorescence when incubated with glycine (p = 0.009) compared to the control. Exposure to sarcosine and DMG also led to a significant increase in fluorescence albeit only after 90 min of incubation (p = 0.03 and p = 0.007, respectively), and not to a level as high as seen after exposure to glycine. In contrast, betaine did not show any influence on polyP concentration under these alkaline incubation conditions.

## Discussion

Bacterial viability can be assessed at different levels: growth capacity, structural integrity and physiological integrity. Culture-based methods, such as the MIC and plate count methods represent the original concept of bacterial viability, solely based on their growth capacity [16]. Our plate count results show a significant linear regression of the CFU after exposure to glycine, sarcosine and DMG (but not betaine), compared to the control (PBS, pH 9.5). This observation, together with the results obtained in previous work [1], indicates that a prolonged exposure to these former compounds induces a progressive loss of ETEC growth capacity under alkaline conditions. Our MIC data only confirmed a growth inhibiting effect for glycine at pH 9.5. However, this latter approach does not provide detailed information on the effects of the compounds at the single cell level, nor on the structural and physiological state of the bacteria.

Essential in bacterial structural integrity is an intact cytoplasmic membrane. We previously described a membrane-damaging effect of glycine, sarcosine and DMG (but not betaine) on stationary phase *E. coli* under alkaline conditions [1]. To ensure on optimal evaluation of the physiological viability parameters described in the present study, we needed to investigate all potential effects on log phase *E. coli*, rather than stationary phase *E. coli*. Our results confirm the loss of membrane integrity, through a nucleic acid flow cytometric staining protocol, and measuring leakage of 260-nm-absorbing material. Both datasets show a significant loss of membrane integrity after 30 min when exposed to glycine, and after 90 min when exposed to sarcosine and DMG. These bacterial populations are still able to grow, as demonstrated by the culture-dependent data. We therefore presume that the initial membrane damage is still reversible, but will progress to irreversible membrane integrity loss upon prolonged exposure (fig. 1c). This leads to a certain heterogeneity in the bacterial population and a final loss of culturability as demonstrated in our previous work [1]. To provide a detailed assessment of bacterial viability and identify any underlying effects of the compounds, a combination of several viability parameters was further examined.

In metabolically active bacteria with intact cytoplasmic membranes, there is typically a difference of electrical potential across the membrane, with a relatively negative intracellular environment compared to the extracellular environment [31]. Changes in membrane potential are considered an early indicator of injury in bacteria [32]. Although a depolarization effect was expected, hyperpolarization has also been reported as an effect associated with bacterial viability loss [5], [19], [33]. Recent studies on this phenomenon suggest that hyperpolarization can occur due to an increase in pH or due to increased movement of ions [34]. More specifically K^+^ diffuses outside the cell through K^+^-channels affecting cellular homeostasis [34]. Spindler et al. [5] state that a non-lethal destabilization of the cytoplasmic membrane can disrupt normal electron flow, resulting in at least a transient hyperpolarization of the cytoplasmic membrane. Hyperpolarization is also defined as a higher negative charge at the intracellular side of the cytoplasmic membrane [35], as shown in fig. 1b. When we consider our compounds in this context, it should be noted that the amine group of glycine, sarcosine and DMG is unprotonated at highly alkaline pH (extracellular) and protonated at a pH near neutrality (intracellular), while the carboxylate group remains negatively charged within this pH range (fig. 1c) [1]. Thus, once intracellular, the amine group becomes protonated at the near neutral cytoplasmic pH. This causes an alkalinization of the cytoplasm and a more negative charge by proton consumption. Of relevance, hyperpolarization can directly comprise membrane integrity [36]. Hyperpolarization has also been associated with the formation of superoxide radicals [5], which are implicated in bacterial killing [37].

The *E. coli* metabolic activity was further evaluated by two complementary parameters: the esterase activity and the total ATP concentration of the bacterial population. Bacterial physiology can only be maintained if the cell is metabolically active and enzymatic reactions, such as those catalyzed by esterases, reflect this activity [38]. No loss of bacterial esterase activity occurred either after exposure to each of the compounds or to alkaline stress alone (control, PBS pH 9.5). Therefore, under these conditions *E. coli* could be considered metabolically viable. Nevertheless, it should be emphasized that while enzyme synthesis requires energy, the esterase enzyme-substrate reaction does not, and this enzymatic process can be considered energy-independent [39]. True assessment of the energetic state of bacteria is possible by the determination of their ATP level. ATP is the universal energy currency of living cells and as such drives numerous energy-consuming reactions e.g. biosyntheses, mechanical motility, transport through membranes and regulatory networks [40]. Indeed, ATP is the most important indicator of the metabolic state and health of cells, including bacteria [40], [41]. The ATP concentration of the bacterial population decreased significantly and very rapidly after exposure to glycine, sarcosine and DMG (but not betaine) at pH 9.5, compared to the alkaline stress control (fig. 1c). This instant depletion cannot be attributed to an extracellular leakage of ATP, as our data present both the intracellular and extracellular ATP concentration.

Two pathways exist in *E. coli* for ATP synthesis: glycolysis and oxidative phosphorylation. The F_o_ F_1_-ATP synthase complex catalyzes the synthesis of ATP from ADP and inorganic phosphate using the electro-chemical gradient of protons generated by respiration during oxidative phosphorylation (fig. 1a) [23]. In addition to ATP synthesis, the respiratory chain has been reported to regulate the cytoplasmic pH in *E. coli* [24]. Krullwich et al. [42] state that when bacterial cells are exposed to conditions which require proton influx into the cytoplasm, such as alkaline stress, the expression of the proton pumping F_o_ F_1_-ATP synthase is elevated in *E. coli*, along with a repression of proton extruding respiratory chain complexes. In line with the latter, our results show a minor, but significant decrease in respiratory activity when the bacteria are exposed to glycine and sarcosine for 15 min. However, this appears to be a transient effect, as respiratory function is already regained at 30 min of incubation.

The F_o_ F_1_-ATP synthase complex is located in the bacterial cytoplasmic membrane and converts the free energy of the proton motive force (PMF) into the universal chemical energy source ATP [43]. Under physiological conditions (i.e. in the absence of alkaline pH stress), protons move from extracellular to the intracellular, allowing ATP generation (Fig. 1a). In *E.* coli, the PMF driving this synthesis consists of both a transmembrane proton concentration gradient (▵pH) and an electrical membrane potential (▵Ψ) component [44]. Both ▵pH and ▵Ψ are influenced by the extracellular pH [22], while the ▵Ψ is additionally modified by several ion membrane transporters (Fig. 1a) [45]. As stated above, the extracellular environment is relatively more acid than the intracellular environment at physiological pH. Under alkaline stress conditions, the situation is reversed which severely influences the PMF [46]. The ▵Ψ increases to compensate for this inverted ▵pH (fig 1b). Intracellular pH homeostasis in an alkaline environment places a high energy demand on the cell. While numerous responses to pH stress are described, the mechanism by which *E. coli* maintains its cytoplasmic pH near neutrality is very complex and remains only partially understood [42], [46]. It is however generally accepted that sustaining this pH homeostasis presents bacteria with a severe bioenergetic challenge. Besides a major influence on bacterial homeostasis, the alkaline conditions also have an important influence the ionization state of glycine, sarcosine and DMG, but not on that of betaine. The ionization state of the trimethylated analogue betaine remains unaffected. In contrast, the amine group of glycine, sarcosine and DMG is protonated at the near neutral cytoplasmic pH, causing an alkalinization of the cytoplasm by proton consumption (fig. 1c). A relevant illustration of this proton scavenging mechanism is provided by Yohannes *et al.* [47], who report that alkaline pH plays a critical role in polyamine stress. These authors state that the accumulation of polyamines is favored when the cytoplasmic pH is lower than the external pH. Under such conditions, the uncharged base entering the cell is protonated in the cytoplasm and its consumption of protons can impair the ongoing pH homeostasis process [7]. This implies that glycine, sarcosine and DMG, but not betaine, could also have the potential to amplify pH stress by scavenging protons within the cytoplasm, thus requiring the cell to spend more ATP to support pH homeostasis (Fig. 1c). This increased metabolic energy requirement could explain the very rapid ATP depletion observed in *E. coli* at alkaline pH after incubation with the presumed proton scavengers glycine, sarcosine and DMG in our *in vitro* model. A depletion of the ATP pool has many detrimental consequences, as energy is required for numerous cellular reactions [39] and can lead to significant membrane damage [19].

Additionally, our results also show an increase in the inorganic polyphosphate pool (polyP) when the bacteria are exposed to glycine, sarcosine and DMG under alkaline conditions. PolyP can be used in response to a wide variety of metabolic needs and plays central roles in many general physiological processes and even as a buffer against alkaline conditions [48]. It is also involved in the response to several different stress situations and is involved in the SOS response, as well as in the stringent response and in RpoS activation [20], [21]. Although the importance of polyP has been reported for various bacterial species, the precise molecular mechanism by which it enacts specific functions, as well as the primary and secondary effects of polyP accumulation, are still not fully understood in even the best characterized bacterial species [49].

In conclusion, we state that exposure to glycine, sarcosine and DMG rapidly induces a marked decrease in ETEC viability under alkaline stress conditions. As to the possible mechanism behind this striking viability loss, several assumptions can be made, which all lead to a final loss of membrane integrity. Our data have shown that *E. coli* ATP depletion and membrane hyperpolarization are the major processes preceding severe membrane damage. We provide strong indications that these can be linked to the intracellular proton scavenging effects of glycine, sarcosine and DMG under alkaline conditions.
